# Theory of Mind Deficit versus Faulty Procedural Memory in Autism Spectrum Disorders

**DOI:** 10.1155/2013/128264

**Published:** 2013-06-04

**Authors:** Miguel Ángel Romero-Munguía

**Affiliations:** Outpatient Service, “Dr. Samuel Ramírez Moreno” Psychiatric Hospital, Health Secretariat, Autopista México-Puebla Km 5.5 Santa Catarina, Tláhuac, 13100 Mexico, DF, Mexico

## Abstract

Individuals with autism spectrum disorders (ASD) have impairments in social interaction, communicative capacity, and behavioral flexibility (core triad). Three major cognitive theories (theory of mind deficit, weak central coherence, and executive dysfunction) seem to explain many of these impairments. Currently, however, the empathizing-systemizing (a newer version of the theory of mind deficit account) and mnesic imbalance theories are the only ones that attempt to explain all these core triadic symptoms of ASD On the other hand, theory of mind deficit in empathizing-systemizing theory is the most influential account for ASD, but its counterpart in the mnesic imbalance theory, faulty procedural memory, seems to occur earlier in development; consequently, this might be a better solution to the problem of the etiology of ASD, if it truly meets the precedence criterion. Hence, in the present paper I review the reasoning in favor of the theory of mind deficit but with a new interpretation based on the mnesic imbalance theory, which posits that faulty procedural memory causes deficits in several cognitive skills, resulting in poor performance in theory of mind tasks.

## 1. Introduction

Autistic disorder is characterized by impairments in reciprocal social interaction, communicative capacity, and repetitive patterns of behavior (core triad) [1]. The term autism spectrum disorders (ASD), which has been used by some mental health professionals as a synonym of pervasive developmental disorder (PDD) [2], implies that these impairments are shared among autistic disorder, Asperger's syndrome, and pervasive developmental disorder not otherwise specified (PDD-NOS) [3]. However, considering that the diagnosis of PDD-NOS does not require the presence of the three diagnostic domains of the autistic triad (social interaction, communicative capacity and behavioral flexibility), the inclusion of PDD-NOS as an ASD has been questioned [4]. This observation is important because it is hard, if not impossible, to make a cognitive theory on disorders that do not share clinical and etiological features [5]. Consequently, I use the term ASD throughout this work to refer to the group comprised only by autistic disorder and Asperger's syndrome, whereas both disorders are mentioned separately when they are not forming a group in some mentioned study in this text.

On the other hand, when Kanner provided the first clinical description of autistic disorder, he also wrote “Their excellent rote memory… It is difficult to know for certain whether the stuffing as such has contributed essentially to the course of the psychopathologic condition” [6]; thus, a cognitive hypothesis was the first one proposed to explain the etiology of autistic disorder. Moreover, a second cognitive hypothesis was established early by Asperger, who surmised that autistic intelligence was an extreme variant of male intelligence. He based his hypothesis on the belief that men were innately more capable of abstract thought than women, whereas women were innately more focused on emotions than men [7]. Nevertheless, neither Kanner's nor Asperger's theory had any influence on the initial development of the three major cognitive theories that have guided research in ASD: theory of mind deficit, weak central coherence, and executive dysfunction [8–10]. Surprisingly, two more recent theories (empathizing-systemizing and mnesic imbalance) make reference to those old hypotheses [11, 12].

The theory of mind deficit account has attempted to explain impairments in social interaction and communicative capacity; the weak central coherence theory seems to explain remarkable abilities of some autistic individuals, and the executive dysfunction theory has been proposed to explain impairments in their behavioral flexibility [13, 14]. In contrast, two theories have attempted to account for the whole autistic triad. Thus, the empathizing-systemizing theory proposes that ASD may be explained by hyperdeveloped systemizing with hypodeveloped empathizing, defining systemizing as drive to analyze or construct systems while empathizing has been defined as drive to infer mental states (theory of mind) and to have an appropriate emotional reaction to them [11, 15]. On the other hand, the mnesic imbalance theory posits that ASD may be explained by faulty procedural memory with relatively preserved declarative memory: defining procedural memory as behavioral algorithms that operate at the unconscious level, while declarative memory is defined as information that is subject to verbal reflection [12, 16].

In the present review article only faulty procedural memory and theory of mind deficit are discussed, whereas relatively preserved declarative memory and hyperdeveloped systemizing will be reviewed next in another work.

## 2. Tests of False Belief

### 2.1. First Study in Autistic Disorder

As mentioned above, the ability to recognize mental states (thoughts and emotions) is one of the two major elements of empathizing. This ability is called theory of mind (mentalizing or mind-reading) [8, 11, 15]; its deficit in autistic disorder was initially tested using the unexpected transfer test of false belief, in which children watch a story about two dolls: one of them (Sally) has a marble, which she puts in a basket and leaves the scene; when she is away, the marble is transferred by the other doll (Anne) from the basket to a box. Then, the experimenter asks the children to infer where Sally will look for the marble. The children pass the test if they point to the basket by taking into account the knowledge (false belief) from Sally. That study found that 85% of typically developing control children passed this test, whereas 80% of children with autistic disorder failed. Thus, its authors surmised that theory of mind deficit is independent from intellectual ability, since the children with autistic disorder had a mean verbal mental age (VMA) as measured by the British Picture Vocabulary Test (BPVT), of 5 years 5 months, while the typically developing control children had a mean chronological age (CA) of 4 years 5 months [8].

### 2.2. Language as an Index of Mental Age

If only vocabulary tests were considered as index of the overall linguistic ability of individuals with ASD in some studies on tests of false belief, such ability could be overestimated, since the picture vocabulary tests adequately estimate verbal mental age in typically developing persons but not in people with ASD because the performance in vocabulary tests is a peak of ability in these individuals [17]. Moreover, some studies have observed impaired grammatical abilities in comparison to the level of lexical abilities in ASD [18, 19]; a study reported lack of significant correlation between grammar and vocabulary in ASD [20]; another study showed slower reaction times to grammatical errors than to lexical errors [21]. These findings are noteworthy because a stronger association has been reported between grammar and test of false belief in children with ASD and mean VMA by the British Picture Vocabulary Scale 2nd edition (BPVS-II) of 7.28 years than in those in control group of children with moderate learning difficulties (MLD) and VMA of 7.58 years by BPVS-II, who in addition had a significantly better performance in tests of theory of mind [22]; also other deficits in communicative function such as pragmatics, reception of gestural language and verbal commands, prosody and discourse comprehension have been found in people with ASD matched for receptive vocabulary with controls, but it is not easy to know whether such deficits are the cause or consequence of theory of mind deficit (mindblindness) or if both traits are caused by a third variable [23–26].

#### 2.2.1. Vocabulary: Lexical Mental Age

Furthermore, the declarative/procedural model assumes that grammar is stored in procedural memory, whereas vocabulary is stored in declarative memory [27]. So, the mnesic imbalance theory may explain grammatical abnormalities in ASD, as well as why some individuals with autistic disorder can repeat many words, phrases, and sentences from their memory, without being able to create expressions appropriate to the context (echolalia) [25, 28], since declarative memory involves consciously recollected information, whereas procedural memory involves unconscious behavioral algorithms [12, 16]. Other deficits in communicative functions (e.g., prosody) in people with ASD also might be due to the lack of simultaneous application of procedural knowledge (simultaneousness) and not just by faulty procedural memory [24, 25], while the relationship between mnesic imbalance and other features of the language (e.g., neologisms) of individuals with ASD has been recently pointed out [29]. So, if there are difficulties in language from individuals with ASD despite their relatively preserved vocabulary, then the poor performance in some theory of mind tasks might be due to those difficulties of language. For this reason, the term “lexical mental age” (LMA) will be used, following other authors [24, 26], throughout this paper instead of VMA when some vocabulary test has been used as an index of mental age.

However, a greater amount of empirical studies is required in individuals with ASD that compare aspects of language that are based on declarative versus procedural memory in order to have conclusive evidence for the mnesic imbalance theory.

### 2.3. Typical Development versus Development in ASD

It should be noted that the majority of typically developing 3-year-old children gives incorrect responses in tests of false belief, such as the unexpected content test of false belief, in which they are shown a tube of Smarties (or M&Ms) and the experimenter asks them what it contains. After they give their answer (candy), they are shown that the tube only contains a pencil. Then, subjects are asked to infer what another child, who has not seen the tube, will think it contains. They pass the test if they respond candy (the false belief). Thus, it has been assumed that 3 year olds have an inability to assign conflicting truth values to propositions despite having the ability to make theory of mind [30]. In contrast, this same deficit in subjects with autistic disorder (CA 13.6; LMA: 6.2) has been interpreted as profound difficulty in making theory of mind [31]. The latter assumption is debatable, since many individuals with autistic disorder who fail in unexpected transfer test of false belief pass nonstandard false belief tasks such as the explicit false belief task in which, for instance, they watch a story about a protagonist (Mary) who wants to find her kitten. It is explicitly mentioned that the pet is in the bedroom and that she thinks her kitten is in the kitchen. Then, they should answer the question: where will Mary look for her kitten [32]? Furthermore, both typically developing 3 year olds and children with autistic disorder significantly improve on tests of false belief when they use thought bubbles to represent mental states [33, 34]. These findings suggest that the difficulty with tests of false belief might be secondary to a poor ability to make inferences. Indeed, a study found that children and adolescents with autistic disorder were impaired relative to LMA-matched controls (7.9 versus 7.46 years) at physical-state counterfactual conditional reasoning, which requires answering questions such as “If there had been no fire, where would Peter be?” but do not understand mental states; the authors of that study suggested that difficulty with standard false belief tasks in autistic disorder might be due to deficits in counterfactual conditional and inferential reasoning, rather than by inadequate theory of mind [32]. On the other hand, it has been reported that the ability to extract the content of a proposition embedded in another proposition (knowledge of complement syntax) significantly predicts the performance on standard false belief tasks [26]. This relationship has been tested in a study in children with ASD utilizing a complement syntax task in which the participants hear stories such as “She said there was a spider in her cereal, but it was really a raisin”; then they respond to the question “What did she say?” The results showed a significantly stronger correlation between complement syntax scores and unexpected transfer (Sally-Anne) test of false belief performance in the ASD group (LMA: 6.87 years) compared with the control group (LMA: 6.37 years), but neither the ASD group (LMA: 6.34 years) nor the control group (LMA: 6.20 years) showed a significant correlation between the unexpected content (Smarties) test of false belief and the complement syntax [35]. In addition, another study mentioned above showed that children with MLD performed significantly better on the Smarties test than on the Sally-Anne test. More specifically, 61% of those children with MLD and inconsistent performance between both tests of false belief failed only the Sally-Anne test whilst passing Smarties test, whereas only 19% of those children with ASD who performed inconsistently the tests showed the same pattern of performance [22]. These different patterns of results deserve to be explained to justify the utilization of both tests in any study. A possible explanation for these results might be found in the way that performance on standard false belief tasks can be improved in typically developing children. For instance, most 3 year olds give correct answers in the unexpected content test of false belief when using a “syntactic method”. That is, the experimenter asks them the same question (what will [name of the other child] think is in the tube?) but with a clause tagged at the end that makes it temporally more specific: What will [name of the other child] think is in the tube before I [experimenter] take the top off [36]? On the other hand, to my knowledge, there is no syntactic method to improve unexpected transfer test of false belief performance, although there is a nonsyntactic method to do that (thought bubbles) [33, 34]. So, any unconscious algorithm (procedural knowledge) similar to the syntactic method might normally be used to resolve unexpected content test of false belief, whereas another unconscious algorithm (procedural knowledge) similar to the thought bubbles would normally be used during unexpected transfer test of false belief, which might explain why the passers and failures in the ASD group obtained equal complement syntax scores than those in the control group to solve unexpected content test of false belief; in contrast, the largest difference in complement syntax scores has been observed between passers and failures, only among the ASD group, to solve unexpected transfer test of false belief [35]. Indeed, this same difference has been observed with the Test for Reception of Grammar (TROG) scores [22]. This greater variation in complement syntax and reception of grammar suggests that these language skills have little true impact on tests of false belief performance; hence, the significant correlation between these language skills and performance in tests of false belief might be an artifact since the ASD groups showed a significantly worse performance relative to comparison groups on tests of false belief, despite the fact that mean performance on complement syntax and reception of grammar were similar in both types of groups, but with a greater variation of these two variables [22, 35].

Finally, counterfactual conditional and inferential reasoning might be considered as a single type of reasoning, called analogical inference, which is linked to procedural memory because it may be done without awareness of the processes that one performs; moreover, the unconscious algorithms that are perhaps normally involved in the solution of tests of false belief might also be analogical inference; consequently, it would be interesting to conduct correlation studies between analogical inference tasks and tests of false belief in people with ASD [37–40].

## 3. Executive Dysfunction

Notwithstanding, overall language skills appear to be associated with overall performance in false belief tasks, as was demonstrated in one of the first studies on executive dysfunction in ASD, in which the Verbal Intellectual Quotient (VIQ) was derived from performance in only three subtests (information, similarities, and vocabulary) of intelligence scales developed by Wechsler. Individuals with ASD were divided into two groups: ASD individuals with or without current autistic language abnormalities, with CA of 11.79 versus 12.38 years. The former group achieved lower VIQ (mean = 75.69) scores than the latter (mean = 92.30). In addition, participants were asked to solve first- (including unexpected content test of false belief) and second-order theory of mind theory tasks [41]. The latter test involves the ability to infer the person's beliefs about another person's beliefs; one example is the story about two children (John and Mary) who see an ice-cream man in the park. John wants ice-cream but does not have money, and the ice-cream man tells John that he will stay the rest of the afternoon in the park, but after John goes home to get money the ice-cream man decides to go and sell ice-cream in the school. Mary knows this entire situation, but after the ice-cream man passes John's house and tells John that he will stay in the school to sell ice-cream. Mary did not know that they had met, because she at that time was still in the park. Then she looks for John in his house and his mother tells her that John has gone to buy an ice-cream. The participants are asked to infer where Mary thinks that John has gone to buy the ice-cream. They pass the test if they respond “the park” (the false belief) and correctly explain why Mary thinks that [42]. On the other hand, in the above mentioned study on executive dysfunction, significant deficits on first- and second-order theory of mind tasks were found only within the ASD group with the lowest VIQ, in comparison to the control group matched for VIQ, whereas the ASD group with the highest VIQ did not show these deficits. However, both groups with ASD had significantly more deficits in executive function tests (Tower of Hanoi; Wisconsin Card Sorting Test) than did control groups. Therefore, the authors concluded that neither executive dysfunction nor theory of mind deficit causes the other, but both might have a common causal factor [10, 41]. Indeed, I have argued that the alterations described by the three major cognitive theories may be caused by imbalance between a faulty procedural memory and a relatively preserved declarative memory [12]. For instance, as previously mentioned, the Tower of Hanoi and the Wisconsin Card Sorting Test have been used to evaluate executive function, but these tests have also been linked to procedural learning [43, 44].

## 4. Theory of Mind Tasks beyond False Belief

Since several individuals with ASD pass tests of false belief, including the second-order false belief task, the majority of adults with Asperger's syndrome (people with ASD and average or above average Intellectual Quotient (IQ), as well as no history of language delay) passes this latter task; researchers have developed more difficult tests such as the strange stories test, the test of faux pas detection, the eyes task, and the Cambridge mindreading face-voice battery in order to show theory of mind deficit in all subjects with ASD [13, 14, 28].

### 4.1. Strange Stories Test

In a study utilizing the strange stories test, which consists of stories where the characters say utterances that are not literally true for various justifications, the participants responded to the questions “Was it true, what X said? Why did X say that?” Justifications given by participants to the “Why” question were scored as either correct or incorrect. Then, correct mental justifications were taken as evidence of theory of mind. The results showed that the ASD group that passed second order false belief tasks (CA 17.7 years; VIQ 95.8) had significantly lower correct mental justifications than control groups. For instance, in a sarcasm story, the mother of Ann cooks the favorite food of Ann, but when the mother brings it to Ann, she is watching TV and does not say thank you. Then the mother of Ann says “That's what I call politeness!” An individual with ASD explained this utterance with the justification that she said it to avoid disturbing her daughter. On the other hand, it is interesting to note the significantly lower number of incorrect mental state justifications on the contrary emotion story from an ASD group (who passed, at least, first-order false belief tasks: CA 20.6 years; VIQ 87.3) relative to children with typical development (CA 8.6 years), while the opposite pattern occurred for most of the other strange stories [45]. A possible explanation is that strange stories as sarcasm and white lie might require the activation of chains of emotional behaviors similar to the chains of motor acts (mirror neurons), but other stories such as the contrary emotion do not. Indeed, it has been observed that in typically developing children, the muscles of the mouth are activated as soon as they move their arm to reach food, as well as during the observation of this same action performed by another individual, whereas, in children with ASD, none of these activations are observed [46]. Therefore, it has been suggested that children with ASD cannot understand the intentions of others because these mirror neurons are not activated [47, 48]. However, when one hears strange stories on persuasion or sarcasm, chains of emotional behaviors (beyond the mirror neurons: chains of motor acts) might be activated to understand the emotions of the protagonists, whereas the strange stories on contrary emotion or lie do not require such activations because the emotions are explicitly mentioned in them [49]. In previous paper I argued that the mirror neurons might be part of the procedural memory since they operate at the unconscious level [12]; such reasoning might be applied to chains of emotional behaviors.

Nevertheless, a study of adults with high functioning autism (HFA), that is, autistic disorder with average or above average IQ, and a history of language delay (CA 30.71 years; VIQ 107.59), as well as adults with Asperger's syndrome (CA 27.77 years; VIQ 110.82), reported the highest number of errors on the stories of double bluff rather than on the other types of stories; it was surmised that the reason for this is that the double bluff story is a third-order theory of mind task [50]. However, the ability to understand double bluff stories does not seem to be associated with the level of performance on theory of mind tasks, since individuals who failed to pass all of the false belief tests obtained the same number of correct mental justifications on the double bluff story than those who passed first order false belief tests [45]. Also it was suggested that the metarepresentation “he *knows *they *think* he will *lie*” indicates second order false belief in double bluff story, but this is similar to metarepresentation “she *knows *they will be *sad* if she does not *want*” in white lie story. However, the latter was easier to understand for group with ASD than the former [45, 50]. This result is in agreement with a study that showed that the ability to respond to new situations (such as the double bluff story) appears to be diminished in individuals with ASD when they have been exposed to the local context (such as false belief tests) [51]; that is, the individuals with ASD had learnt in tests of false belief that the protagonists cannot know a location if they have not seen or heard that [8, 42], which might be a restrictive procedural knowledge that makes difficult to understand double bluff stories, whereas in pretend and figure of speech stories do not [45, 49, 50]. So, correlation studies are necessary among tests of false belief, each item of the strange stories test, and analogical inference tasks to verify these hypotheses.

### 4.2. Test of Faux Pas Detection

Another test of theory of mind is the test of faux pas detection, which evaluates the ability of individuals to recognize when a speaker says something that the listener might not want to know. The results of a study demonstrated that children with ASD (CA 144.0 months; LMA 159.0 months) perform poorly on this test; in addition, although some of them could recognize the faux pas, still they could not inhibit them [52]. This latter finding could be due to any failure in chains of emotional behaviors.

In sum, strange stories test and test of faux pas detection show hypothetical scenarios to individuals who should infer the appropriate mental states. However, another measurable variable in this type of tests may be the reaction time, which significantly decreased with age between adolescence and adulthood in typically developing people [53, 54]. Moreover, the reduction of reaction time during repeated sequences utilizing tests such as the Serial Response Time Task (SRTT) is an index of procedural learning; the SRTT has indicated significant impairments in procedural learning from people with ASD [16, 55]. Thus, studies comparing reaction times between theory of mind and procedural learning tasks would be useful to test the mnesic imbalance theory.

### 4.3. Eyes Task

The eyes task (reading the mind in the eyes task) consists of showing the participants photographs of the eye region, through which they should infer the mental state of people just from the information in those photographs. A study demonstrated that adults with ASD (CA 28.6 years; IQ 105.31) are impaired in their ability to infer mental states from photographs of persons' eyes compared with two control groups, one of adults with Tourette's syndrome (CA 27.77 years; IQ 103.5) and another group of typically developing adults (CA 30.0 years) [56]. The deficits already mentioned, such as lack of simultaneousness, analogical inference, or chains of emotional behaviors, do not appear to be appropriate to explain the results from the eyes task, but procedural learning has been linked to another skill; this is, so-called perceptual categorization, which can be achieved through two types of category-learning tasks: rulebased and information-integration. In rulebased tasks, the categories can be acquired through declarative learning, whereas in information-integration tasks, the categories can be acquired via procedural learning [57]. Afterward, if participants can assign every photograph of the eye region to any category of mental state, then the ability to solve this task must have been acquired through procedural learning, since it is difficult (if not impossible) to verbally explain (i.e., by declarative memory) what characteristics of each photograph can justify its assignment to certain category of mental state; consequently, impaired performance in the eyes task in the group with ASD might be the result of lack of unconscious algorithms [12]. So, even some typically developing adults performed the eyes task worse than some individuals with ASD [56]. Then, a revised version of the eyes test was developed which increased the number of photographs from 25 to 36, as well as from two to four answer choices for each photograph: thereby, these changes improved the reliability of the test [58]. Still, the original version of the eyes test showed theory of mid deficit, whereas a basic emotion recognition task did not [56]. This result could be because the basic emotions can be easily explained by declarative knowledge; for example, the emoticons of happiness {:-)}, sadness {:-(}, and surprise {:-O} only require a simple change in the shape of the mouth, whereas the photographs of the eye region on complex emotion such as worry, insistence, or uneasy cannot be distinguished from each other by any explicit feature. Thus, the mnesic imbalance theory also might explain the results of other studies, for instance, one measured the visual fixation time in adolescents and young adults with autistic disorder (CA 15.4 years; VIQ 101.3) on eye, mouth, body, and object regions when they watched movies, finding a significant positive correlation between the percentage of fixation time on the mouth region and level of social competence in this group, whereas the fixation time on the eye region did not correlate with their social skills. In addition, the study showed that they focused twice as much on the mouth region and two times less on the eye region, in comparison with the control group (CA 17.9 years; VIQ 102.5) [59].

These data are in agreement with another study which reported that children with autistic disorder (CA 83.7 months; IQ 82.2) who were asked to identify four basic emotions on photographs of faces had a performance above chance when they viewed the lower face, whereas their performance was within chance levels when they viewed the upper face alone. In addition, the author of that study proposed the hypothesis that an automatic system (procedural) orients us to the upper face to understand the emotional states of others, whereas an intentional system (declarative) is linked to the lower face [60].

### 4.4. Cambridge Mindreading Face-Voice

The Cambridge mindreading face-voice is a battery that uses several emotion concepts via video and audio in order for the participants to show their ability to recognize complex emotions in the face and in the voice. In a study utilizing this battery, the results demonstrated that a group of adults with Asperger's syndrome (CA 30.2 years; VIQ 114.4) was significantly impaired relative to control adults (CA 27.1 years; VIQ 118.47) [61]. According to the mnesic imbalance theory, this result might be explained by lower development of perceptual categorization and chains of emotional behaviors [12].

In several studies have been observed significant differences in brain activity in theory of mind tasks between adolescence and adulthood; for instance, typically developing adolescents (aged 9–17 years) showed activation of the anterior cingulate cortex during viewing of fearful faces relative to neutral faces, whereas typically developing adults (age 25–36 years) did not [62]. Similarly, a study showed higher activity in anterior cingulate cortex of men with HFA (ages 20–33 years) in comparison with the control group [63]. In addition, using the antisaccade task (a test of inhibition) in individuals with typical development (ages 12 to 23 years) showed that inhibition (as index of executive function) has the most important correlation with affective theory of mind (emotion inferences) than updating and shifting (others two executive functions) [64]. These data are in accordance with my hypothesis of higher activity in anterior cingulate cortex by overload for declarative memory in people with HFA [16], while such overload can cause very significant inhibitory errors [65].

Several tests using film scenes, such as the awkward moments test [66], the movie for assessment of social cognition [67], and the “reading the mind in film” task [68, 69], have been developed in order to assess theory of mind skills of children and adults with ASD during tasks more similar to genuine social settings than in previous tests. As expected, in all experiments, the ASD groups scored significantly lower on these film tasks, compared to control groups.

During another experiment, a group with ASD (CA 30.29; VIQ 116.29) and a control group (CA 30.21; VIQ 116.93) watched film clips of a test named the Moral Dilemmas Film Task and were asked to write about what happened in each clip. Then, the narratives given by participants were scored for total number of references to objects and mental states, as well as of protagonists to whom mental states were attributed. The results showed that the ASD group had a slightly lower number of references to objects and a significantly lower number of references to mental states relative to the control group. However, there was no statistically significant difference between the two groups in the number of individuals who spontaneously applied second order theory of mind to at least one protagonist (*P* = 0.085); besides, only the ASD group had a significant correlation between the number of references to mental states and the VIQ but not the Empathy Quotient (EQ), a questionnaire used to assess empathizing (theory of mind; appropriate emotional reaction) [70]. These data suggest that the ASD group generated complex inferences of mental states despite not believing that they had good theory of mind on others.

This discrepancy between ability and intention to make theory of mind might be explained by an interesting model which suggests that theory of mind can be divided into affective (emotion inferences) and cognitive theory of mind (belief inferences); the latter has to integrate with empathy (the drive to establish theory of mind) and emotional contagion for making affective theory of mind [71]. In particular, the empathy might be the drive to infer mental states in the Moral Dilemmas Film Task, which does not require participants to correctly make theory of mind on film characters [70]. Furthermore, the affective theory of mind may be based on perceptual categorization and chains of emotional behaviors, while cognitive theory of mind may be based on analogical inference. Also in the mnesic imbalance theory, the empathy might be the drive to show procedural knowledge; the simultaneousness would facilitate a better demonstration of the procedural knowledge.

## 5. Conclusions

This paper argues that a faulty procedural memory causes deficits in several cognitive skills such as simultaneousness, analogical inference, chains of emotional behaviors, and perceptual categorization and that all these deficits may explain the theory of mind deficit measured by the tests of false belief (analogical inference), the strange stories test and the test of faux pas detection (analogical inference; chains of emotional behaviors), the eyes task, and the Cambridge mindreading face-voice (perceptual categorization). In other words, although several studies are necessary in order to verify the hypotheses on the faulty procedural memory, they seem to explain impairments in social interaction from people with ASD, but these cannot explain the other features of individuals with ASD: insistence on sameness, idiosyncratic behaviors, fixated interests, abnormal reactions to sensory stimuli, and special isolated skills. Consequently, the mnesic imbalance theory requires a second factor named “relatively preserved declarative memory” [12], which will be discussed next in another review article, although I have already published preliminary works on that topic [29, 72].
